# Supplementary material for: Modulation of Gut Microbiome and Autism Symptoms of ASD Children Supplemented with Biological Response Modifier: A Randomized, Double-Blinded, Placebo-Controlled Pilot Study
Source: Nutrients. 2024 Jun 21;16(13):1988. doi: 10.3390/nu16131988 (PMC11243103; doi:10.3390/nu16131988)
Supplement: Supplementary file 1 [file nutrients-16-01988-s001.zip › Table S2.pdf]

**Table S2: Number of lost or acquired OTUs within individual bacterial families before and after supplementation of ASD children.** The OTUs composition within individual bacterial families underwent significant changes during the 3-month trial.

| Family | No. of OTU - JUVENIL | | No. of OTU - PLACEBO | |
|---|---|---|---|---|
| | newly identified | disappearing | newly identified | disappearing |
| ***Methanobacteriacea*** | 0 | 0 | 0 | 0 |
| ***Actinomycetaceae*** | 1 | 0 | 1 | 2 |
| ***Bifidobacteriaceae*** | 0 | 4 | 1 | 3 |
| ***Atopobiaceae*** | 0 | 0 | 0 | 0 |
| ***Coriobacteriaceae*** | 0 | 3 | 1 | 1 |
| ***Eggerthellaceae*** | 2 | 2 | 1 | 6 |
| ***Bacteroidaceae*** | 9 | 6 | 7 | 11 |
| ***Barnesiellaceae*** | 2 | 0 | 1 | 4 |
| ***Marinifilaceae*** | 3 | 4 | 3 | 7 |
| ***Muribaculaceae*** | 3 | 0 | 0 | 0 |
| ***Porphyromonadaceae*** | 0 | 2 | 1 | 0 |
| ***Prevotellaceae*** | 3 | 4 | 4 | 3 |
| ***Rickenellaceae*** | 6 | 2 | 2 | 4 |
| ***Tannerellaceae*** | 3 | 3 | 5 | 5 |
| ***Flavobacteriaceae*** | 0 | 2 | 0 | 0 |
| ***Campylobacteriaceae*** | 2 | 0 | 0 | 1 |
| ***Gastranaerophilales*** | 2 | 0 | 1 | 0 |
| ***Desulfovibrionaceae*** | 4 | 2 | 1 | 1 |
| ***Erysipelatoclostridiaceae*** | 2 | 0 | 1 | 1 |
| ***Erysipelotrichaceae*** | 4 | 5 | 3 | 3 |
| ***Enterococcaceae*** | 0 | 1 | 0 | 1 |
| ***Lactobacillaceae*** | 1 | 1 | 1 | 4 |
| ***Leuconostocaceae*** | 1 | 0 | 0 | 1 |
| ***Streptococcaceae*** | 2 | 3 | 1 | 8 |
| ***Clostridia*_UCG-014** | 2 | 3 | 9 | 3 |
| ***Clostridia*_vadinBB60_group** | 0 | 4 | 6 | 4 |
| ***Clostridiaceae*** | 1 | 2 | 0 | 2 |
| ***Christensenellaceae*** | 13 | 5 | 9 | 15 |
| ***Defluviitaleaceae*** | 0 | 1 | 0 | 1 |
| ***Lachnospiraceae*** | 34 | 57 | 15 | 93 |
| ***Monoglobaceae*** | 0 | 1 | 1 | 2 |
| ***[Eubacterium]_coprostanoligenes*_group** | 5 | 1 | 2 | 3 |
| ***Butyricicoccaceae*** | 2 | 0 | 0 | 6 |
| ***Oscillospiraceae*** | 11 | 18 | 11 | 25 |
| ***Ruminococcaceae*** | 18 | 27 | 21 | 35 |
| **UCG-010** | 3 | 1 | 1 | 4 |
| ***Peptococcaceae*** | 2 | 2 | 3 | 4 |
| ***Anaerovoracaceae*** | 2 | 1 | 4 | 5 |
| ***Peptostreptococcaceae*** | 1 | 1 | 0 | 0 |
| ***Peptostreptococcales-Tissierellales*** | 2 | 1 | 4 | 1 |
| ***Acidamonococcaceae*** | 1 | 0 | 0 | 0 |
| ***Selenomonadaceae*** | 1 | 0 | 2 | 1 |
| ***Veillonellaceae*** | 1 | 9 | 0 | 10 |
| ***Fusobacteriaceae*** | 0 | 1 | 0 | 2 |
| ***Saccharimonadaceae*** | 0 | 1 | 1 | 2 |
| ***Aeromonadaceae*** | 0 | 1 | 0 | 1 |
| ***Oxalobacteriaceae*** | 0 | 1 | 0 | 0 |
| ***Sutterellaceae*** | 0 | 8 | 3 | 3 |
| ***Enterobacteriaceae*** | 0 | 8 | 3 | 14 |
| ***Morganellaceae*** | 1 | 0 | 0 | 0 |
| ***Pasteurellaceae*** | 2 | 2 | 1 | 2 |
| **vadinBE97** | 0 | 0 | 1 | 1 |
| ***Victivallaceae*** | 0 | 0 | 0 | 0 |
| ***Akkermansiaceae*** | 0 | 0 | 0 | 0 |

**Table S2: Number of lost or acquired OTUs within individual bacterial families before and after supplementation of ASD children.** The OTUs composition within individual bacterial families underwent significant changes during the 3-month trial.

Only those OTUs that were not present in even one ASD patient before the administration of Juvenil or placebo were included in this table, and conversely, the given OTU appeared completely newly in at least one patient after the end of supplementation.
